# Influencing Mechanism of Nod-Like Receptor Protein 3 Inflammasome Activation in A375 Cell Activity in Human Cutaneous Malignant Melanoma

**DOI:** 10.1155/2022/7420330

**Published:** 2022-07-15

**Authors:** Akebaier Sulaiman, Jin Lv, Junwei Fan, Reyila Abuliezi, Qian Zhang, Xuefeng Wan

**Affiliations:** Department of Dermatology, The First Affiliated Hospital of Xinjiang Medical University, Urumqi 830054, Xinjiang Uyghur Autonomous Region, China

## Abstract

This work was to investigate mechanism by which mir-22 targeting nod-like receptor protein 3 (NLRP3) inflammasome affected activity of human skin malignant melanoma (MM) A375 cells. Twenty-four mice were rolled into a control group (Group X) and an experimental group (Group Y) randomly. Without treatment in Group X, Group Y established MM model. After cell transfection, the mice were divided into group A (blank group), group B (negative group), group C (miR-22 mimics group), group D (miR-22 inhibitor group), and group E (miR-22 inhibitor+siNLRP3 group). The results were summarized as follows. The level of miR-22 mRNA in Group Y was obviously lower than that in Group X, and levels of NLRP3 and caspase-1 mRNA and NLRP3 and caspase-1 protein in Group Y were greatly higher than those in Group X (*P* < 0.05). The mRNA levels of miR-22 mRNA in group C were much higher in contrast to those in group A, and the mRNA levels of NLRP3 and caspase-1 were lower. The contrast results in group D and group A were the opposite, *P* < 0.05. The levels of NLRP3 and caspase-1 proteins in group C were greatly elevated, and those in group D were decreased compared with those in group A (*P* < 0.05). Therefore, miR-22 may target and inhibit the activation of the NLRP3 inflammasome to reduce the activity of cutaneous malignant melanoma A375 cells.

## 1. Introduction

Malignant melanoma (MM) is referred to as evil black, which is a malignant tumor caused by melanin cells. MM is featured with strong capacities of invasion and transfer and usually appears on the skin and mucosa where melanin cells are abundant [1]. According to incomplete research data, the incidence of MM accounts for 1% to 2% among that of all malignant tumor diseases. It is a common cutaneous malignant tumor following squamous cell carcinoma and basal cell carcinoma [2]. MM is one of the fastest growing malignant tumors with an increasing mortality year by year. The statistics and analysis of the incidence of MM by multiple domestic and foreign research institutes demonstrate that the incidence of MM among white-skin population is high, mainly in Europe and America. In contrast, it is relatively lower in the Asian-Pacific region dominated by the yellow race [3, 4]. Besides, it is proposed in many studies that the incidence and mortality of MM among males are both higher than those among females in the regions with different levels of economic development to some degree. From the perspective of clinical attention, MM is still one of the diseases detrimental to the health of all Chinese people because of its high level of malignancy, rapid metastasis, inadequate ability to diagnose early symptoms, and insufficient understanding of MM by doctors and patients [5]. Due to the characteristics of high malignancy, rapid metastasis of MM disease, and insufficient accuracy of early diagnosis, treatment is difficult and affects the quality of life and long-term prognosis of patients.

Inflammasome is one of the essential components involved in nonspecific immunity. At present, the study on nod-like receptor protein 3 (NLRP3) inflammasome is the most complete and clear [6, 7]. NLRP3 inflammasome is a polyprotein complex formed by the interaction of NLRP3 molecules, pro-cysteinyl aspartate specific proteinase-1 (pro-caspase-1) and apoptosis-associated speck-like protein containing a CARD (ASC) [8]. Currently, the specific activation mechanism of NLRP3 inflammasome is still explored and researched. Generally, it is believed that NLRP3 inflammasome can be activated by two steps, including initiation (step 1) and activation (step 2). There are many signals activating NLRP3 inflammasome, while its activation mechanism is still unclear [9]. Some researchers find a new way of inflammasome activation, which is different from traditional ways of NLRP3 inflammasome activation. The new way is the activation caused by stimulating human monocyte NLRP3 inflammasome through the signal pathway TLR4-TRIF-RIPK1-FADD-CASP8 without depending on two signals by lipopolysaccharide (LPS) [10]. It promotes the activation of caspase-1, which is cleaved to recombinant human interleukin-1*β* proteins (IL-1*β*) and IL-18 precursors to generate bioactive IL-1*β* and IL-18, which are released outside cells [11]. In addition, IL-1*β* induces the synthesis of IL-1*β* precursors through signal pathway IL-1RI-MyD88-NF-*κ*B to form a positive feedback loop [12].

In the process of the incidence and development of human cutaneous MM, NLRP3 promotes the developmental progress of melanoma by affecting host tumor immunity and promoting the proliferation and differentiation of A375 cells in melanoma as well as regulating its microenvironment [13]. Relevant research reports reveal that NLRP3 is the targeted gene of micro ribonucleic acid-22 (miR-22). Pathway miR-22/NLRP3 exerts a vital role in the apoptosis of endothelial cells. miR-22 can be targeted to decrease the NLRP3 level to inhibit the activation of downstream caspase-1 and the cleavage of IL-1*β* and IL-18 precursors to mature bodies. As a result, the endothelial apoptosis mediated by inflammatory reactions can be alleviated [14, 15]. In this work, it was supposed that miR-22/NLRP3 could regulate the biological behaviors of A375 cells in human cutaneous MM to influence the molecular mechanism of the A375 cells in MM. In this work, the primary melanoma A375 cell lines were cultured in vitro, and different transfection protocols were innovatively used to explore the mechanism of the activation of miR-22 targeting the NLRP3 inflammasome on the viability of human skin malignant melanoma A375 cells, to find the target of skin malignant melanoma targeted drug research and development and provide a sustainable theoretical basis for the search for accurate, efficient, and reliable targeted drugs for the treatment of malignant melanoma.

## 2. Materials and Methods

### 2.1. Animals for Experiment

Table 1 displays relevant information about the included animals for the experiment.

Under specific pathogen-free (SPF) condition, 24 mice were fed at about 26°C and the relative humidity ranged between 45% and 55%. Besides, the feeding environment should be ventilated every day, the food fed to 24 mice was sterile standard fixed mixture, and the drinking water was autoclave distilled water. 24 mice were fed in separate cages (6 in each cage), and they could eat and drink freely. The ratio of illumination to darkness each day was 1 : 1 (12 hours for each). The experiment was conducted at the Experimental Animal Center Laboratory. After the animal experiment, animals were treated by professionals based on humanitarian principles.

### 2.2. Experimental Reagents and Apparatuses

Table 2 displays the experimental reagents and apparatuses needed in the experimental process.

According to animal mouse models, the most suitable instruments and equipment that were used most commonly in clinical practice in recent years were selected, which enabled the experimental operations and results to be more similar to clinical results, laying a good foundation for clinical application and promotion of the research in the future.

### 2.3. Culture of A375 Cells in MM

According to the cell culture instructions provided by Shanghai Yaji Biotechnology Co., Ltd., A375 primary cell strain in melanoma was cultured in vitro. After disinfection and sterilization, the corresponding preparation before the treatment was prepared in the sterile cell laboratory. After that, complete medium (containing 10% FBS) was prepared by FBS and DMEM with potential of hydrogen (pH) of 7.4 in the ratio of 1 : 9. Besides, a certain amount of complete medium was extracted with a 5 mL pipette. In a sterile flask with appropriate size, melanoma A375 cells were cultured. Next, the flask was placed in an incubator with saturated humidity, 37°C, and 5% carbon dioxide concentration. According to the growth of cells, liquid exchange, digestion and passage, freezing storage, and experimental treatment were carried out irregularly on a super clean bench.

### 2.4. Construction of Mouse Models and Experimental Methods

Random number table method was utilized to divide 24 BALB mice into 2 groups, including a control group (Group Y) and an experimental group (Group X) (12 in each). The mice in Group X received no special treatment. The melanoma A375 cells cultured in vitro were inoculated subcutaneous tissues of the mice in Group Y to establish MM mouse models. The injection was performed once every 15 days, and the experiment lasted for 3 months.

The cell transfection and grouping methods were as follows [16]. In a sterile environment, some parts of mouse subcutaneous melanoma tissues were sectioned and taken to extract RNA, and the remaining tissues were placed and washed with phosphate buffer solution (PBS) containing double antibody. After that, they were centrifugated at 300∗*g* for 10 minutes to dispose of the supernatant. The culture medium was 20% FBS DEME-F12 with the temperature of 37°C, moderate humidity, and 5% carbon dioxide in air environment. The cells to be transfected were implanted onto a 12-well plate at the density of 2 × 10^5^/cm^2^ 1 day before the transfection. When the cell fusion rate reached 60%~80%, miR-22 mimics transfection and the corresponding negative control were carried out according to the transfection kit instructions. As a result, group A (blank group without transfected cells), group B (negative group with the transfection of irrelevant sequences), group C (miR-22 mimics group with the transfection of miR-22 overexpression products), group D (miR-22 inhibitor group with the transfection of miR-22 inhibitors), and group E (miR-22 inhibitor+siNLRP3 group with the transfection of miR-22 and inactivation of NLRP3 genes) were formed. After the verification of transfection efficiency, the subsequent experimental steps were implemented.

The mRNA levels of miR-22, NLRP3, and caspase-1 in mouse melanoma subcutaneous tissues were detected by using real-time fluorescence quantitative PCR method [17]. NLRP3 and caspase-1 protein expression levels [18] were detected by using Western blot. Besides, ELISA kits were used to detect the concentration of IL-1*β* and IL-18.

### 2.5. Statistical Analysis

SPSS 22.0 was selected to analyze and process the included data. Measurement data were given in the form of mean ± deviation ($\bar{x}\pm s$). One-way analysis of variance (ANOVA) was utilized for the comparison among multiple groups. Least-significant difference (LSD) was used for pairwise comparison. *T* test was adopted to compare the measurement data of subjects in two. Besides, *P* < 0.05 indicated that the differences showed statistical meaning.

## 3. Results

### 3.1. General Data on Cutaneous MM

**Figure 1:**
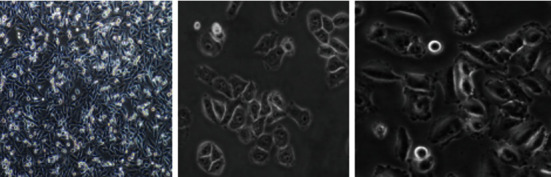
Electron microscopic images of cutaneous MM A375 cells. (a–c) The magnification factors were ×10, ×20, and ×40, respectively.

**Figure 2:**
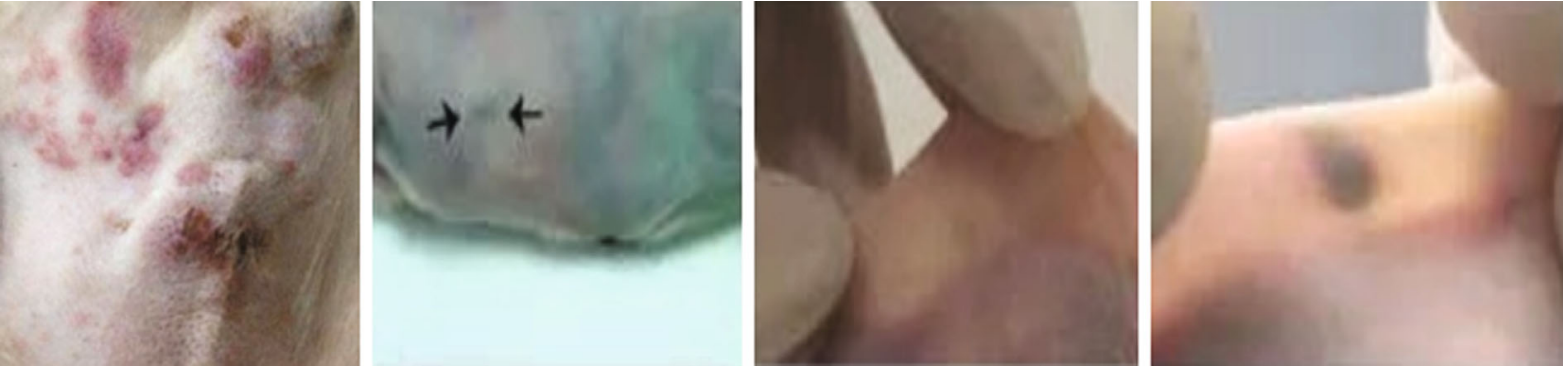
Images of mouse cutaneous MM models.

Figure 1 shows the electron microscopic images of cutaneous MM A375 cells, and Figure 2 displays the images of mouse cutaneous MM models.

### 3.2. Expression Levels of miR-22, NLRP3, and Caspase-1 mRNA in Experimental and Control Groups

**Figure 3:**
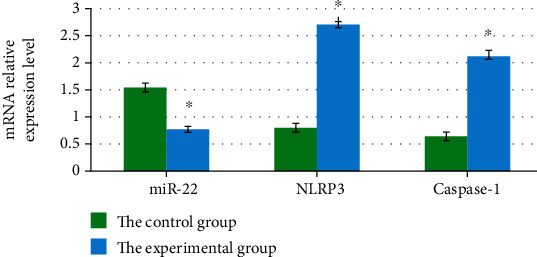
Levels of mRNA in miR-22, NLRP3, and caspase-1 (∗ indicated that the comparison with the control group showed *P* < 0.05).

In subcutaneous tissues of the included mice, mRNA levels of miR-22, NLRP3, and caspase-1 in Group X were 1.55 ± 0.08, 0.80 ± 0.07, and 0.67 ± 0.06, respectively, while those in Group Y were 0.77 ± 0.04, 2.68 ± 0.06, and 2.13 ± 0.09, respectively. The above results demonstrated that miR-22 mRNA level in mice in Group Y (mice with MM) was greatly decreased and the levels of NLRP3 and caspase-1 mRNA in Group Y were both remarkably higher than those in Group X (normal mice). *P* < 0.05 suggested that the differences showed statistical meaning, as Figure 3 displays.

### 3.3. Expression Levels of NLRP3 and Caspase-1 Proteins in Experimental Group and Control Group

**Figure 4:**
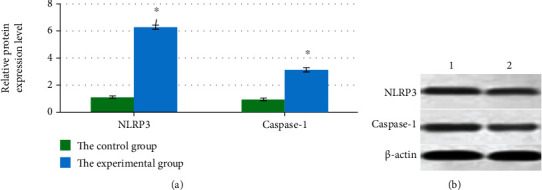
Levels of NLRP3 and caspase-1 proteins in subcutaneous tissues. (a) The protein expression level of NLRP3 and caspase-1. (b) The protein band of NLRP3 and caspase-1; 1 and 2 referred to the experimental group and the control group, respectively. ∗ indicated that the comparison with the control group showed *P* < 0.05.

In subcutaneous tissues in the included mice, the levels of NLRP3 and caspase-1 proteins of mice in Group X were 1.21 ± 0.10 and 1.05 ± 0.07, respectively, while those in Group Y were 6.45 ± 0.13 and 3.23 ± 0.09, respectively. It revealed that the levels of NLRP3 and caspase-1 proteins in Group Y were both higher obviously compared to those in Group X. *P* < 0.05 indicated that differences showed statistical meaning, as Figure 4 illustrates. As demonstrated in Figure 4(b), the brightness of NLRP3 and caspase-1 protein bands in the experimental group was higher than that in the control group.

### 3.4. Expression Levels of miR-22, NLRP3, and Caspase-1 mRNA in Groups A–E

**Figure 5:**
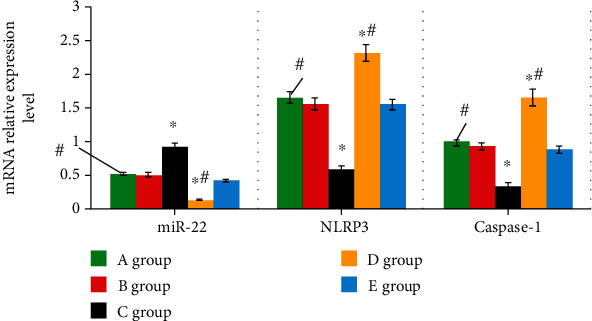
Levels of miR-22, NLRP3, and caspase-1 mRNA. ∗ indicated that the comparison with group A showed *P* < 0.05. # indicated the comparison with group C showed *P* < 0.05. Among the above items, group A was the blank group, group B was the negative group, group C was the miR-22 mimics group, group D was the miR-22 inhibitor group, and group E was the miR-22 inhibitor+siNLRP3 group.

miR-22 in groups A~E showed the levels of 0.52 ± 0.06, 0.51 ± 0.04, 0.93 ± 0.09, 0.13 ± 0.03, and 0.42 ± 0.06, respectively. NLRP3 level in each group was 1.66 ± 0.04, 1.57 ± 0.10, 0.58 ± 0.06, 2.33 ± 0.09, and 1.56 ± 0.07, respectively; caspase-1 mRNA levels in all groups were 0.99 ± 0.03, 0.93 ± 0.06, 0.35 ± 0.05, 1.66 ± 0.05, and 0.89 ± 0.05, respectively. The above results demonstrated that, compared with group A, the level of miR-22 mRNA in group C was much higher, and the levels of NLRP3 and caspase-1 mRNA were obviously lower (*P* < 0.05). The miR-22 mRNA in group D showed an obviously lower level than the level in group A, while the NLRP3 and caspase-1 mRNA were significantly higher (*P* < 0.05), showing statistically significant difference, as Figure 5 illustrates.

### 3.5. Expression Levels of NLRP3 and Caspase-1 Proteins in Groups A–E

**Figure 6:**
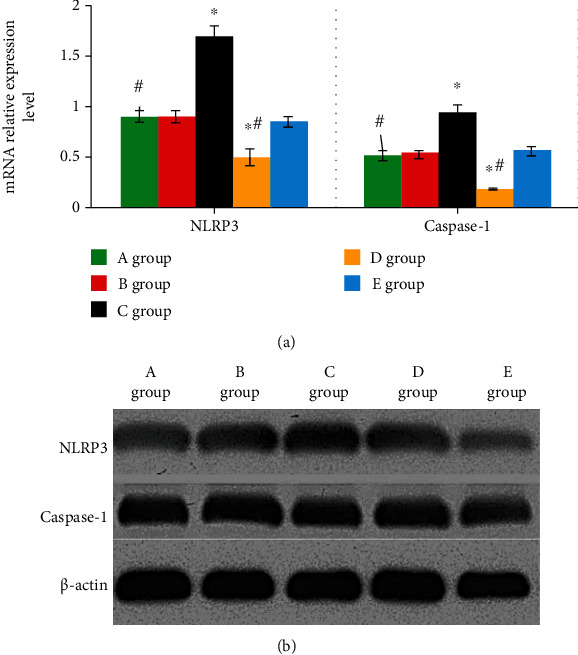
Levels of NLRP3 and caspase-1 proteins. (a) The protein expression level of NLRP3 and caspase-1. (b) The protein band of NLRP3 and caspase-1. ∗ indicated that the comparison with group A showed *P* < 0.05. # indicated the comparison with group C showed *P* < 0.05. Among the above items, group A was the blank group, group B was the negative group, group C was the miR-22 mimics group, group D was the miR-22 inhibitor group, and group E was the miR-22 inhibitor+siNLRP3 group.

The levels of NLRP3 and caspase-1 proteins were 0.92 ± 0.06 and 0.55 ± 0.08, were 0.93 ± 0.06 and 0.55 ± 0.03, were 1.74 ± 0.04 and 0.99 ± 0.06, were 0.47 ± 0.09 and 0.20 ± 0.09, and were 0.88 ± 0.08 and 0.58 ± 0.08 in groups A~D, respectively. The above results demonstrated that the levels of NLRP3 and caspase-1 proteins in group C were both higher than the values in group A. In addition, the expression levels of NLRP3 and caspase-1 proteins in group D were both remarkably lower compared with those in group A. *P* < 0.05 indicated that the differences showed statistical meaning, as Figure 6 illustrates.  Figure 6(b) illustrates that the brightness of NLRP3 and caspase-1 protein bands in group C was the highest, followed by groups B and E, while those in groups A and D were the lowest.

### 3.6. Comparison of Levels of Inflammatory Factors

Among the above items, group A was the blank group, group B was the negative group, group C was the miR-22 mimics group, group D was the miR-22 inhibitor group, and group E was the miR-22 inhibitor+siNLRP3 group.

**Figure 7:**
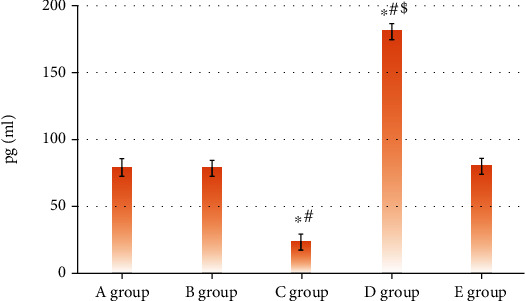
Levels of IL-18. (∗, #, and $ indicated the comparison with group A, group B, and group C showed *P* < 0.05, respectively.)

**Figure 8:**
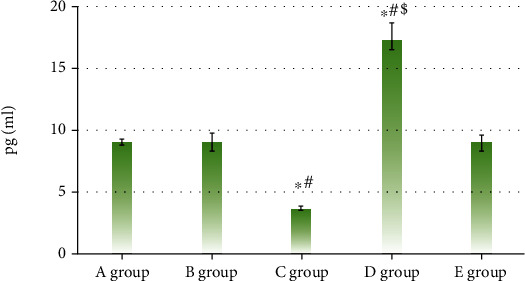
Expression levels of IL-1*β* in each group. (∗, #, and $ indicated the comparison with group A, group B, and group C showed *P* < 0.05, respectively.)

IL-18 in five groups showed the levels of 9.14 ± 6.22, 79.11 ± 5.15, 25.14 ± 3.05, 180.35 ± 5.24, and 80.54 ± 4.84, respectively. The levels of IL-1*β* in the five groups were 9.03 ± 0.27, 9.04 ± 0.67, 3.69 ± 0.13, 17.11 ± 1.57, and 9.03 ± 0.54, respectively. The above results demonstrated that levels of IL-18 and IL-1*β* in group C were observably lower than those in groups A and B (*P* < 0.05). The comparison of the expressions of IL-18 and IL-1*β* among groups D, A, and B was opposite. *P* < 0.05 indicated that the differences showed statistical meaning, as Figures 7 and 8 illustrate.

## 4. Discussion

At present, surgery is still the main and decisive therapeutic method of early melanoma, while it rarely cures advanced melanoma [19]. The application of immunotherapy and targeted therapy prolongs patients' survival, which revolutionizes the clinical history of the disease. However, most patients become resistant to targeted drugs after the treatment for several months. More and more evidence indicate that miRNA is the key factor of the incidence of drug resistance [20, 21]. The research results show that miR-22 inhibited the activating NLRP3 inflammasome by targeting, reduced the activity of A375 cells in mouse cutaneous MM tumors, and regulated the expression of inflammatory factors, including IL-1*β* and IL-18, which strengthened the protection effects of mouse subcutaneous tissues.

To screen drugs quickly and efficiently in the body, some scholars investigated the method of constructing mouse melanoma models with the primary cells in mouse melanoma solid tumor tissues [22]. Melanoma B16 is a tumor that spontaneously occurs at the ear root of C57BL/6 mice. The injection of melanin tumor cells into receptor mice by subcutaneous, intravenous, or peritoneal inoculation could construct the corresponding melanin tumor animal models [23]. Primary A375 cell strains in melanin tumors were cultured in vitro and inoculated at mouse subcutaneous tissues to construct melanoma mouse models. The results of the research indicated that miR-22 expression level of the MM group was obviously reduced compared with that in Group X. In contrast, the levels of NLRP3 and caspase-1 were obviously increased.

One of the current inflammasomes with the clearest structure and function is NLRP3 inflammasome, which is a key member of oligomerized nucleotide binding domain-like receptor (NLR) family. It consists of NLRP3, caspase-1, and ASC [24]. In cutaneous MM mouse models, NLRP3 inflammasome was activated and overexpressed in A375 cells in MM. Its expression was correlated with the severity of melanoma to some extent [25]. The expression of miR-22 mRNA in group C was notably higher, while the levels of NLRP3 and caspase-1 mRNA were opposite in contrast to group A (*P* < 0.05). The results of the comparison between group D and group A were opposite (*P* < 0.05). The differences showed statistical meaning. The above results demonstrated the overexpression of miR-22 could reduce the activity of A375 cells in mice with cutaneous MM mice and the apoptosis rate of subcutaneous cells. To be brief, miR-22 could target NLRP3 to reduce the activity of melanoma A375 cells by targeting.

In some literature, it was documented that NLRP3 inflammasome is very important in the influencing mechanism on activity of human cutaneous MM A375 cells [26]. The activated NLRP3 inflammasome could induce the activation of caspase-1 to promote the maturity and secretion of IL-1*β* and IL-18 precursors [27], which was similar to the results of the research. The experiment indicated that the levels of IL-18 and IL-1*β* in group C were lower greatly than the values in groups A and B (*P* < 0.05). In contrast, the comparison of the levels of IL-18 and IL-1*β* in groups D, A, and B was opposite. *P* < 0.05 indicated that the differences showed statistical meaning. Currently, there were 11 known IL-1 family members. The combination of IL-1*β* and the receptor 1 of IL-1 was one of the important inflammatory signals [28]. Hence, it was concluded that miR-22 decreased the expressions of IL-1*β* and IL-18 by targeting NLRP3 genes. As a result, the inflammatory injury of subcutaneous cells was alleviated.

## 5. Conclusion

The nude mouse tumor-bearing models were established, aimed at analyzing the influencing mechanism of the activation of miR-22-targeted NLRP3 inflammasome on activity of human cutaneous MM A375 cells. It was found out that miR-22 could cause adverse effect on activating NLRP3 inflammasome by targeting to reduce the cell apoptosis in subcutaneous tissues of cutaneous MM mouse models and regulate IL-18 and IL-1*β*. As a result, the inflammatory injury of subcutaneous cells was mitigated. The finding might provide a new research direction for the clinical treatment of cutaneous MM. However, the finding was still not supported by clinical data. Therefore, the effect and molecular mechanism of miR-22 in the clinical treatment of human cutaneous MM needed to be further researched.

## Figures and Tables

**Table 1:** Relevant information about experimental animals.

Variety and strain	Week-age	Gender	Number	Body mass	Qualification number	Sale organization
BALB/c mice	4–7 weeks	Female	24	16-24 g	/	The Experimental Animal Center

**Table 2:** Experimental reagents and apparatuses.

Experimental reagents and apparatuses	Manufacturers
Olympus ckx53 inverted fluorescence microscope	Shanghai Danding International Trading Co., Ltd.
Carbon dioxide incubator	Shanghai Bluepard Instruments Co., Ltd.
Fetal bovine serum (FBS)	Wolcavi (Beijing) Biotechnology Co., Ltd.
Dulbecco's modification of eagle's medium (DMEM)	Wuhan Procell Life Science & Technology Co., Ltd.
Enzyme-linked immunosorbent assay (ELISA) kit	Wuhan Saipei Biotechnology Co., Ltd.
Polymerase chain reaction (PCR) instrument	Beijing Cycloud Biotechnology Co., Ltd.

## Data Availability

The data used to support the findings of this study are included within the article.
